# Which intraperitoneal insufflation pressure should be used for less postoperative pain in transperitoneal laparoscopic urologic surgeries?

**DOI:** 10.1590/S1677-5538.IBJU.2016.0366

**Published:** 2017

**Authors:** Ali Akkoc, Ramazan Topaktas, Cemil Aydin, Selcuk Altin, Reha Girgin, Omer Faruk Yagli, Aykut Bugra Sentürk, Ahmet Metin

**Affiliations:** 1 Department of Urology, Gazi Yasargil Training and Research Hospital, Diyarbakir, Turkey;; 2 Department of Urology, Kartal Yavuz Selim State Hospital, Istanbul, Turkey;; 3 Department of Urology, Hitit University, Training and Research Hospital, Corum, Turkey;; 4 Department of Urology, Abant Izzet Baysal University Faculty of Medicine, Bolu, Turkey

**Keywords:** Pain, Postoperative, Pneumoperitoneum, Laparoscopy, Urology

## Abstract

**Purpose:**

To determine whether using different intraperitoneal insufflation pressures for transperitoneal laparoscopic urologic surgeries decreases postoperative pain.

**Materials and Methods:**

76 patients who underwent transperitoneal laparoscopic upper urinary tract surgery at different insufflation pressures were allocated into the following groups: 10mmHg (group I, n=24), 12mmHg (group II, n=25) and 14mmHg (group III, n=27). These patients were compared according to age, gender, body mass index (BMI), type and duration of surgery, intraoperative bleeding volume, postoperative pain score and length of hospital stay. A visual analog scale (VAS) was used for postoperative pain.

**Results:**

Demographic characteristics, mean age, gender, BMI and type of surgeries were statistically similar among the groups. The mean operation time was higher in group I than group II and group III but this was not statistically significant (P=0.810). The mean intraoperative bleeding volume was significantly higher in group I compared with group II and group III (P=0.030 and P=0.006). The mean length of postoperative hospital stays was statistically similar among the groups (P=0.849). The mean VAS score at 6h was significantly reduced in group I compared with group III (P=0.011). At 12h, the mean VAS score was significantly reduced in group I compared with group II and group III (P=0.009 and P<0.001). There was no significant difference in the mean VAS scores at 24h among three groups (P=0.920).

**Conclusion:**

Lower insufflation pressures are associated with lower postoperative pain scores in the early postoperative period.

## INTRODUCTION

Laparoscopic urologic surgeries have proven to be safe and effective compared with open surgery, offering the benefits of decreased blood loss, less postoperative pain, shorter hospital stay, rapid convalescence and earlier return to normal activities and work, smaller incisions and improved cosmetic effect (1-4). Although laparoscopic surgery reduces postoperative pain (POP), POP is still substantial and constitutes the main clinical problem after laparoscopic urologic procedures.

We surgeons always aim to inprove the quality of care given to patients. One of the strategies studied for less POP over the last years is to lower artificial intraperitoneal pressure level during laparoscopic procedure. Normal and low laparoscopic insufflation pressure are defined as 12-15 and 5-7mmHg, respectively (5). It has been shown that the use of lower pressure pneumoperitoneum reduces the adverse hemodynamic effects of laparoscopic surgery (6, 7). However, its effect on POP remains controversial.

We could find no study addressing the relationship between insufflation pressure and POP in transperitoneal or retroperitoneal laparoscopic urologic surgical procedures. This prospective study was aimed to compare POP in patients underwent transperitoneal laparoscopic upper urinary tract surgery (TLUS) at different insufflation pressures.

## MATERIALS AND METHODS

We had included in this prospective study a total of 78 patients submitted to TLUS between July 2013 and March 2016 for various upper urinary tract pathologies including atrophic or hydronephrotic nonfunctioning kidney, renal cyst, ureteral stone, renal pelvic stone and ureteropelvic junction obstruction. The patients were allocated into three different intraperitoneal insufflation pressure groups. But two patients submitted to laparoscopic simple nephrectomy were excluded from the study because we had to increase intraperitoneal pressure for management of intraoperative bleeding. The operations were performed in 10mmHg and 14mmHg pressures. There were 24 patients in group I (10mmHg), 25 patients in group II (12mmHg), and 27 patients in group III (14mmHg). All laparoscopic procedures were performed by four surgeons. Approval was obtained from the local ethics committee, and written informed consent was obtained from all participants.

Exclusion criteria were pediatric population, oncology cases (due to the need of an additional incision to remove specimen), uncontrolled diseases such as severe hypertension, diabetes mellitus and asthma, neurologic diseases, chronic pain patients, prior abdominal surgery, a history of renal surgery or a solitary renal unit and American Society of Anaesthesiology (ASA) grade 3 or more.

In all cases, the same general anesthesia protocol was used. After induction of anesthesia, a nasogastric tube and a urinary catheter were inserted. All patients were placed in a modified (70 degree) lateral decubitus position with the umblicus over the break in the Table. Pneumoperitoneum was induced in all groups using a Veress needle and CO_2_ was insufflated at a rate of 2L/min until intraabdominal pressures of 10, 12 and 14mmHg were reached. Intraperitoneal insufflation at these pressures was held constant by automatic regulation of the CO_2_ inflow. Firstly, an 11mm trocar was placed, the abdomen was inspected by a 0º, 10mm rigid laparoscope for any injury due to Veress needle or port placements. Then, another 11mm trocar and 5mm trocar were used and these trocars were inserted under direct vision. When necessary, an additional 5mm fourth trocar was selectively used for proper exposure or traction. The surgical procedures were performed according to techniques used in transperitoneal laparoscopy by the types of surgery. Small specimens were retrieved through the 11mm trocar without trocar removal. Large benign specimens were removed after the specimen in the entrapment bag was fragmented by use of scissors without extension of the incision at the end of procedure. A double J stent was used intraoperatively in the twenty patients who were submitted to ureterolithotomy, pyelolithotomy and pyeloplasty. In all cases, residual CO_2_ in peritoneum was evacuated at the end of the procedure by compressing the abdomen. We routinely placed a drainage tube through the lower trocar. After the laparoscopic surgery was completed, all patients were injected with 5mL of 5mg/mL bupivacaine (Marcaine 0.5%, AstraZeneca, Istanbul, Turkey) at all trocar areas.

All patients were routinely prescribed postoperative analgesia, with 20mg tenoxicam (Oksamen L, Mustafa Nevzat, Istanbul, Turkey) administered intravenously at 6 and 18 hours, postoperatively. After 24 hours, the same analgesic was administered to all patients if required. All of the patients were prescribed an oral analgesic drug for use as needed for pain after discharge.

The following study parameters of patients were recorded: age, gender, BMI, type and duration of surgery, intraoperative bleeding volume, postoperative pain score and length of hospital stay. The operation time was calculated from the first trocar insertion to the last trocar extraction. Evaluation of pain was performed postoperatively at 6, 12 and 24 hours. We asked patients to disregard localized and sharp pain around the port incision to exclude parietal pain. The patients were instructed by the physician to complete the visual analogue scale (VAS), ranging from 0 to 10 (0, no pain; 10, the most severe pain), to evaluate any diffuse, dull aching pains in the abdomen or shoulder, representing visceral and referred visceral pains.

All data were analyzed statistically using one-way analysis of variance (ANOVA) followed by the Bonferroni test for multiple comparisons, using the Statistical Package for Social Sciences (SPSS) software for Windows, version 15.0 (SPSS Inc., Chicago, IL, USA). The data are expressed as means±standard deviations (SD). P value of <05 was considered statistically significant.

## RESULTS

During a 33-month period, we included in the study 76 patients who were submitted to TLUS including simple nephrectomy (LSN, n=28), renal cyst decortication (LRCD, n=28), ureterolithotomy (LUL, n=8), pyelolithotomy (LPL, n=6) and pyeloplasty (LPP, n=6). The mean age, gender, BMI and type of surgeries were statistically similar among the groups. The characteristics of the patients and types of surgeries are shown on Table-1. The mean intraoperative bleeding volumes were 115.42±49.87, 85.20±34.70, and 79.25±34.30mL in group I, group II, and group III, respectively, and significantly higher in group I compared with group II and group III (P=0.030 and P=0.006). It was higher in group II than group III but this was not statistically significant (P=1.000) (Table-2). The mean operation time was higher in group I than group II and group III but this was not statistically significant (P=0.810). The mean length of postoperative hospital stays were similar among groups (P=0.849) (Table-2).

**Table 1:** Characteristics of the patients and types of surgeries.

	Group 1 (N=24)	Group 2 (N=25)	Group 3 (N=27)	P Value
Age (years)	37.17±14.75	37.96±14.14	39.00±14.33	0.901
BMI (kg/m^2^)	25.25±2.80	25.00±3.05	25.40±3.37	0.893
**Gender**				
(Male: Female)	13:11	11:14	13:14	0.782
**Type of surgery**				
LRCD	9	9	10	0.990
LSN	9	9	10	
LUL	2	3	3	
LPL	2	2	2	
LPP	2	2	2	

**Table 2:** Postoperative pain scores, perioperative and postoperative parameters.

	Group 1 (N=24)	Group 2 (N=25)	Group 3 (N=27)	P Value
VAS (6h)	4.13±1.12	4.88±1.24	5.14±1.26	0.011^a^
VAS (12h)	2.75±0.73	3.52±1.01	3.74±0.85	<0.001^b^
VAS (24h)	1.95±0.75	2.04±0.74	2.00±0.62	0.920
Operation time (min)	111.17±56.17	102.40±50.65	103.00±51.70	0.810
Intraoperative blood loss (mL)	115.42±49.87	85.20±34.70	79.25±34.30	0.004^c^
Hospital stay (days)	3.50±2.20	3.88±2.68	3.67±2.07	0.849

^a^Group I < Group III p=0.011.

^b^Group I < Group II p=0.009, Group I < Group III p<0.001.

^c^Group I > Group II p=0.030, Group I > Group III p=0.006.

The VAS scores at 6h were 4.13±1.12, 4.88±1.24, and 5.14±1.26 in group I, group II, and group III, respectively, and significantly reduced in group I compared with group III (P=0.011). At 12h, the mean VAS scores were 2.75±0.73, 3.52±1.01, and 3.74±0.85 in group I, group II and, group III, respectively and significantly reduced in group I compared with group II and group III (P=0.009 and P<0.001). There was no significant difference in the VAS scores at 24h among three groups (P=0.920) (Table-2). No patients required any additional analgesic agents for POP management.

## DISCUSSION

Laparoscopy based on refinements in technology and instrumentation developed rather slowly and lately in urology and was adopted from gynecologists and general surgery, so initially it has been based on the transperitoneal approach (8). Since the introduction of laparoscopy in urologic practice, the clinical outcomes of laparoscopic surgery have shown decreased peri- and postoperative morbidity and mortality, shorter hospitalization and convalescence times, smaller incisions and improved cosmesis, and reduced POP compared with open surgery.

Patients in need of upper tract intervention for renal cyst, nonfunctioning kidney, ureteropelvic junction obstruction, or renal pelvic and ureteral stone or for oncological purposes may benefit from those advantages provided by TLUS. Although the duration of hospital stay and recovery time tend to be shorter than that after open surgery, POP is one of the most common complaints and still causes considerable discomfort and increased stress response following laparoscopic urologic procedures.

The types of POP in laparoscopic surgeries are deep intraabdominal pain (visceral pain), incisional pain (parietal pain) and shoulder pain (referred visceral pain) (9, 10). Although visceral pain may also depend on the extent of intraabdominal surgery, incisional pain is related to the number and size of access devices and also to the technique of incision closure and drainage. The reason of shoulder pain is not clear, but it is commonly assumed that the continual stretching of the peritoneum during and after the pneumoperitoneum is responsible. Clinically, incisional and deep abdominal pain dominate over shoulder pain (5). There are several causes of pain following laparoscopic surgery due to the effect of CO_2_ pneumoperitoneum, peritoneal stretching, diaphragmatic irritation, diaphragmatic injury and shoulder abduction during surgery (11, 12). Pneumoperitoneum affects the visceral pain component and theoretically, a low pressure should cause less pain than a high pressure (9) but this issue is still controversial.

Topcu and colleagues evaluated POP following gynecologic laparoscopy in a prospective randomized trial using three different intraabdominal pressures (8, 12, 15mmHg) (13). They showed a positive correlation between the VAS score following laparoscopic surgery and intraperitoneal insufflation pressure values. In the late postoperative period at 12h and 24h, they detected significantly lower pain score in the low pressure group. Additionally, in the early postoperative period at 6h, the VAS score was lower in the low pressure group, but this difference was not statistically significant. In our study, the VAS scores at 6h were significantly reduced with 10mmHg pressure group compared with 14mmHg pressure group. At 12h, the VAS scores were significantly reduced in 10mmHg pressure group compared with 12 and 14mmHg pressure groups. There was no significant difference in the VAS scores at 24h among three groups.

In a prospective randomized double blind trial using 9 or 13mmHg intraabdominal pressure during laparoscopic cholecystectomy, it was reported that the low pressure pneumoperitoneum did not increase the duration of surgery, the frequency and intensity of shoulder tip pain were significantly lower in the low pressure group, and that the dose requirement for analgesic drugs was significantly lower in the low pressure group (14). In our study, there was no significant difference in the operation times and length of postoperative hospital stays among three different insufflation groups, and no patients required any additional analgesic agents for pain management.

In another study, Joshipura et al. reported that the use of low pressure had significant advantages for pulmonary function preservation, POP, analgesic usage, and hospital stay compared with the use of high pressure in pneumoperitoneum during laparoscopic cholecystectomy (15). Similarly, Wallace et al. compared 7.5 and 15mmHg intraabdominal pressures for laparoscopic cholecystectomy and reported that after operation the low pressure group had significantly less pain, better preservation of pulmonary function and shorter hospitalization (16). Some studies, however, have shown that the pressure levels did not affect pain scores. Celik and colleagues compared low (8mmHg), standard (12mmHg) and high-pressure (15mmHg) for laparoscopic cholecystectomy and reported that intraabdominal pressure has no effect on postoperative visceral pain, but has effect on duration of anesthesia and operation (17). Another study demonstrated no difference in low pressure and standard pressure pneumoperitoneum in the outcomes of laparoscopic cholecystectomy. And routine use of lower pressure pneumoperitoneum in laparoscopic cholecystectomy would not be recommended unless in selected straightforward cases (18). Similarly, Perrakis et al. reported that there was no difference in postoperative abdominal pain and analgesic consumption between low (8mmHg) and high (15mmHg) intraabdominal pressure groups (9). The reduction of intraabdominal pressure did not reduce POP.

Logically, total volume of intraperitoneal insufflations of CO_2_ during laparoscopy may be associated with pain in patients submitted to laparoscopic urologic procedures. Total amount of CO_2_ insufflated during laparosopic urologic procedures was not recorded in our study. We think that it was the limitation of our study. The number of patients are limited in the study due to the large number of exclusion criteria. This is the other limitation of the study.

Vilos and colleagues reported that the intraperitoneal pressure was correlated positively with BMI (19). It was suggested the use of low pressure in patients with higher BMIs. In our study, there was no statistical difference in BMI among the groups.

In the literature, various methods have experienced to reduce POP after laparoscopic surgical procedures. The pulmonary recruitment maneuver, intraperitoneal normal saline infusion and using low pressure pneumoperitoneum were found to reduce POP (11, 12, 20, 21). Additionally, intraperitoneal administration of local anesthetics or some analgesics or combination of these; periportal local anesthetic injection; or combined periportal and intraperitoneal administration of local anesthetic were efficient in reducing POP in laparoscopic procedures (22-26). However, some studies reported that periportal, intraperitoneal or combined periportal and intraperitoneal administration of local anesthetic did not influence POP after laparoscopic surgery (27, 28). The majority of studies have been performed on patients who underwent laparoscopic cholecystectomy and laparoscopic gynecological procedures. There is no study on this issue in laparoscopic urologic surgery.

In our study, increased hemorrhage volume and reduced POP were detected in the low pressure group. We found a relationship between lower pressure pneumoperitoneum and less pain, particularly during the early postoperative period. However, there was no significant difference in the pain scores in the late postoperative period, postoperative hospital stays and duration of surgery among the groups. On the basis of those findings, the widespread use of lower pressure should be considered for POP control and patient comfort. According to the recommendation of the European Association for Endoscopic Surgery, a rational approach could be to employ minimum pneumoperitoneum pressure that allows adequate exposure of the surgical field (5).

## CONCLUSIONS

According to our study, however, lower insufflation pressure may result in more increased hemorrhage; but it is associated with less postoperative pain scores in the early postoperative period. Additionally, use of lower pneumoperitoneum pressure in laparoscopy is important for not only postoperative pain but also intraoperative and postoperative other complications of laparoscopy such as subcutaneous emphysema, acidosis, cardiac arrhythmia, gas embolism, pneumothorax. We think that employing minimum intraperitonel insufflation pressure that allows adequate exposure of the surgical field in laparoscopic urologic surgeries is seen as a logical strategy.
